# *In vivo* multi-modality tracking of the regenerative effects of the human induced pluripotent stem cell-derived cardiomyocytes (iCMs)

**DOI:** 10.1186/1532-429X-17-S1-Q119

**Published:** 2015-02-03

**Authors:** Morteza Mahmoudi, Eric Rulifson, Atsushi Tachibana, Mouer Wang, Joseph C Wu, Phillip Yang

**Affiliations:** 1 Cardiovascular Medicine, Stanford University, Palo Alto, CA, USA

## Background

*In vivo* multi-modality cellular and molecular imaging of the engrafted iCMs is necessary to characterize the engraftment and the regional effects on the viability of the injured myocardium. Zinc finger nuclease (ZFN)-mediated integration of the reporter gene into the AAVS1 locus in the iCMs and manganese enhanced MRI (MEMRI) should allow precise *in vivo* detection of myocardial regeneration.

## Methods

In order to have multi-modal *in vivo* imaging of the stem cells, ZFN technology was employed to integrate a triple fusion RG (firefly luciferase (BLI), red fluorescent protein (RFP), and herpes simplex virus thymidine kinase (PET)) into the AAVS1 locus of iPSCs. iPSCs monoclonal lines were then differentiated into iCMs, under chemically defined conditions using CHIR (4 µM) and C59 (2 µM) small molecules to modulate the Wnt pathway activity. The produced iCMs were then fully characterized and injected into mice and tracked with several techniques.

## Results

**Figure 1:**
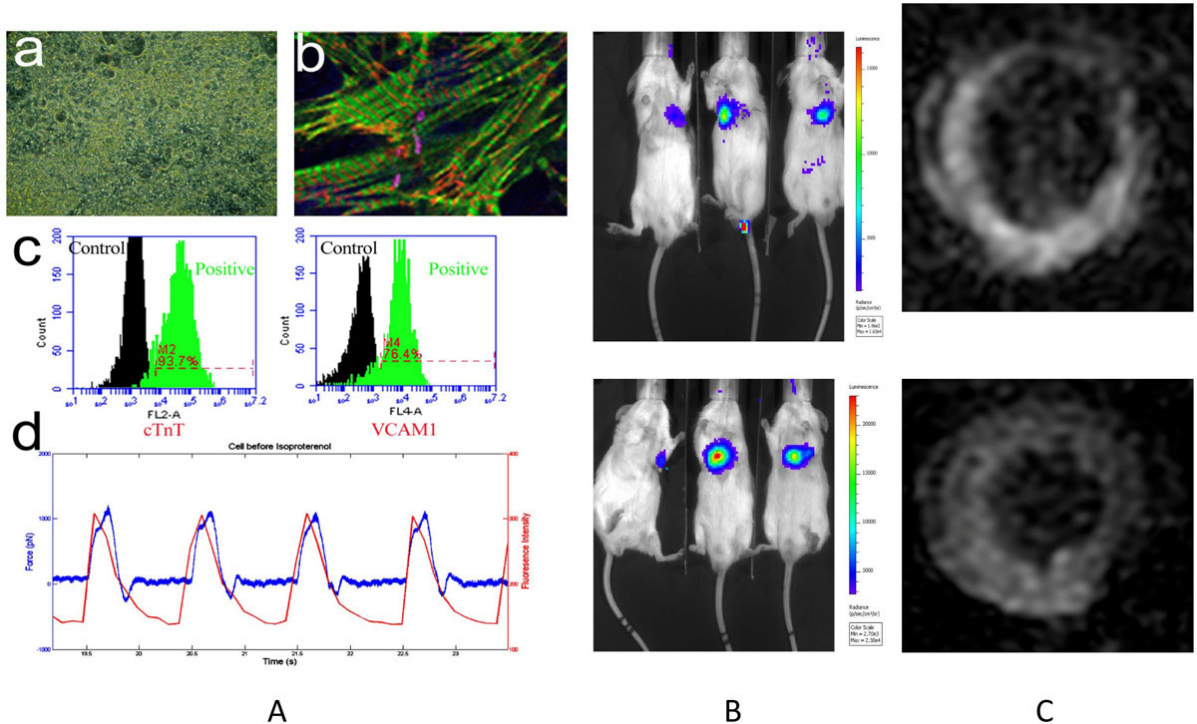
A): a) Optical microscopy of the human pluripotent cell-derived cardiomyocytes (iCMs); b) Confocal image of the iCMs immunolabeled for alpha-Actinitn (green), cardiac TroponinT (red), Connexin43 (violet) and nuclei (blue); c) flow cytometry results showing the existence of cTnT and VCAM1 positive iCMs; d) spontaneous atomic force microscopy (AFM) measurement and calcuim flux in iCMs B) Bioluminescence of RG Luciferase activity from human cells in heart at week 4 after transplant. B) BLI signal of the mice after 2 weeks (top panel) and 4 weeks (bottom panel) of iCM injection. C) MEMRI images of iCMs injection (after 2 (top panel) and 4 weeks (bottom panel)) for myocardial infarct.

Co-localization of cardiac phenotype and striated morphology, flow cytometry measurement of 93% cardiac troponin (cTNT) associated with 75% VCAM and 49% CIRPA cell surface markers, the atomic force measurement with calcium flux were observed, which confirmed normal function of the iCMs (Fig. 1A). The iCMs were, then, transplanted into murine myocardial injury model. Two and four weeks post-injury, left ventricular ejection fraction (LVEF) was better preserved in the iCM-treated *vs.* PBS-treated mice (week 2: 33.1±4.4% *vs.* 26.1±7.9%, p<0.05 and at week 4: 35.1±8.4% *vs.* 16.1±0.2%, p<0.05). MEMRI also demonstrated increased % myocardial viability by iCMs at week 2 measured 67.3±13.1%, which decreased slightly by -2.3±0.3% at week 4 while PBS treated cases conferred significant decrease -17.4% at week 4 (p<0.05). Finally, the mean BLI signal, indicating iCM engraftment, demonstrated on days 14 (n=3) is 2.1±0.6 x10^3^p/s/cm^2/^sr and days 28 (n=3) is 4.2±2.2 x10^3^p/s/cm^2/^sr. Robust *in vivo* expression of ZFN-edited BLI signal of iCM engraftment and sustained MEMRI signal of myocardial viability over 4-week duration demonstrated regeneration of the injured myocardium (Figs. 1B and 1C).

## Conclusions

This study demonstrates the successful use of *in vivo* multi-modality imaging to confirm the regeneration of the injured myocardium. ZFN technology enables targeted site-specific insertion of RGs and MEMRI allows direct evaluation of myocardial viability. These imaging capabilities will allow clinical translation of iCMs.

## Funding

NIH/NHLBI UM1 (HL-12-026).

